# Exploring Sn_x_Ti_1−x_O_2_ Solid Solutions Grown onto Graphene Oxide (GO) as Selective Toluene Gas Sensors

**DOI:** 10.3390/nano10040761

**Published:** 2020-04-15

**Authors:** Eleonora Pargoletti, Simone Verga, Gian Luca Chiarello, Mariangela Longhi, Giuseppina Cerrato, Alessia Giordana, Giuseppe Cappelletti

**Affiliations:** 1 Dipartimento di Chimica, Università degli Studi di Milano, Via Golgi 19, 20133 Milan, Italy; simone.verga@studenti.unimi.it (S.V.); gianluca.chiarello@unimi.it (G.L.C.); mariangela.longhi@unimi.it (M.L.); 2 Consorzio Interuniversitario per la Scienza e Tecnologia dei Materiali (INSTM), Via Giusti 9, 50121 Firenze, Italy; giuseppina.cerrato@unito.it (G.C.); alessia.giordana@unito.it (A.G.); 3 Dipartimento di Chimica & NIS, Università degli Studi di Torino, Via P. Giuria 7, 10125 Turin, Italy

**Keywords:** metal oxide solid solutions, graphene oxide, chemoresistor, volatile organic compounds, sensitivity, selectivity, room temperature sensing

## Abstract

The major drawback of oxide-based sensors is the lack of selectivity. In this context, Sn_x_Ti_1−x_O_2_/graphene oxide (GO)-based materials were synthesized via a simple hydrothermal route, varying the titanium content in the tin dioxide matrix. Then, toluene and acetone gas sensing performances of the as-prepared sensors were systematically investigated. Specifically, by using 32:1 SnO_2_/GO and 32:1 TiO_2_/GO, a greater selectivity towards acetone analyte, also at room temperature, was obtained even at ppb level. However, solid solutions possessing a higher content of tin relative to titanium (as 32:1 Sn_0.55_Ti_0.45_O_2_/GO) exhibited higher selectivity towards bigger and non-polar molecules (such as toluene) at 350 °C, rather than acetone. A deep experimental investigation of structural (XRPD and Raman), morphological (SEM, TEM, BET surface area and pores volume) and surface (XPS analyses) properties allowed us to give a feasible explanation of the different selectivity. Moreover, by exploiting the UV light, the lowest operating temperature to obtain a significant and reliable signal was 250 °C, keeping the greater selectivity to the toluene analyte. Hence, the feasibility of tuning the chemical selectivity by engineering the relative amount of SnO_2_ and TiO_2_ is a promising feature that may guide the future development of miniaturized chemoresistors.

## 1. Introduction

A perhaps endless list of compounds falls under the acronym VOCs, which stands for Volatile Organic Compounds; these share a common feature: they are volatile at ambient temperature. Their molecular nature varies (alcohols, ketones, hydrocarbons and so on) as well as their origin, e.g., emissions from industrial facilities [1,2], commercial products [3], plants [2], animals and human beings as well [4,5]. Thus, with the increasing of environmental concerns [6,7], taking into account the correlations between a specific VOCs and human diseases [8,9,10], the monitoring of these compounds has become mandatory. In particular, it has been recently demonstrated that abnormal concentrations of toluene in the human’s breath could be a potential symptom for lung cancer disease [11]. The exhaled toluene level in lung cancer patients is known to be approximately of hundreds of ppb, which is more than three times greater its concentration in healthy people’s breath (around 20–30 ppb) [12].

Metal oxide semiconductors (MOS) such as ZnO [13,14], SnO_2_ [15,16] and TiO_2_ [17,18] exhibit remarkable sensitivity towards a wide range of both reducing and oxidizing gases. However, their major drawback is their lack of selectivity in the presence of several interfering species. Indeed, most MOS-based gas sensors and, especially those based on the conductivity change upon interaction with gases, are non-specific and, above all, they are usually active at temperatures above 200 °C [19,20]. For instance, small polar molecules are better detected than volatile hydrocarbons, and similar VOCs still give identical responses [21,22]. However, the latter issue can be partially solved by adopting different approaches, such as (i) the synthesis of 3-dimensional hierarchical, mesoporous or hollow networks with enhanced surface activity [23,24,25]; (ii) the engineering of nano-heterojunctions that prompt significant changes in the potential barrier height upon exposure to different analytes [26,27,28]; (iii) the doping or decoration with noble metal nanoparticles having a diverse catalytic effect towards a specific VOC molecule; and (iv) the statistical elaboration of data obtained by multiple sensor arrays [29,30]. Hence, in this context, scientific research is focusing attention on the preparation of novel materials able to sense VOCs at room temperature, thus reducing the energy consumption, reaching high sensitivity (of ppb concentration) and a good selectivity [31,32,33]. For instance, Quan et al. [23] have recently pointed out the hierarchical flower-like Pt-doped 3D porous SnO_2_ possesses excellent selectivity for acetone molecules, showing a good response down to 50 ppb at a relatively high temperature of 153 °C. Besides, 3D hierarchical hollow SnO_2_/ZnO hetero-nanofibers have been described as potential acetone selective sensors thanks to the occurrence of an n-n type junction at the interface between ZnO and SnO_2_, thus resulting in an enhanced electronic transport [26]. Nevertheless, also in this case, the operating temperature with the highest response is 350 °C [26]. Moreover, Kim et al. [34] reported exceptional toluene sensing properties of novel SnO_2_/ZnO core–shell nanowires, functionalized with Pt nanoparticles, showing an excellent R_air_/R_analyte_ intensity response to 100 ppb at 300 °C. Remarkably, as sticks out, the majority of the most promising reported sensor materials achieve the best performances only at high operating temperatures, especially above 250–300 °C [26,34].

In this context and with our promising results already reported on the synergistic effect between p-type MOS and n-type graphene oxide (GO), especially in reducing the operating temperature [27,35], we believe that the Sn_x_Ti_1−x_O_2_/GO solid solution system may be an efficient active material with even improved selectivity. Indeed, there are already several papers [36,37,38,39,40] dealing with both synthesis and successful application of SnO_2_-TiO_2_ mixed oxides as either photocatalysts or sensing materials. Tricoli et al. [37] reported the preparation of a SnO_2_–TiO_2_ solid solution with limited cross-sensitivity towards humidity, thanks to the replacement of some tin cations by titanium ones. Chung et al. [41] suggested that the enhancement of their gas sensing performances relies on the Schottky barrier formation at the grain boundaries of well-formed SnO_2_ particles embedded in a finely dispersed TiO_2_ matrix.

Hence, starting from these previous studies, novel composite materials, based on a combination of SnO_2_, TiO_2_ and GO, are presented herein. Notably, the effect of the substitution of tin by titanium cations has been fully elucidated on structural, surface, morphological and optical points of view. Finally, toluene (i.e., big and non-polar molecule) sensing has been deeply investigated as a function of titanium content in the solid solutions, both at 350 °C and at lower operating temperatures, in the latter case by simultaneously exploiting the UV light. Thus, we found that toluene concentrations of 100 ppb can be detected. Then, the best-performing samples were tested also towards acetone molecules, evidencing that SnO_2_/GO and TiO_2_/GO are more selective towards smaller and polar molecules. Conversely, the solid solutions Sn_x_Ti_1−x_O_2_/GO can detect, to a greater extent, bigger and lower polar analytes, such as toluene species. Moreover, they can be exploited down to 250 °C, while 32:1 SnO_2_/GO and TiO_2_/GO also proved to be very promising at room temperature. Finally, we demonstrated that these optimal nanocomposite structures provide excellent chemical responses, showcasing their applicability as selective room-temperature VOC chemoresistors.

## 2. Materials and Methods

All the chemicals were of reagent-grade purity and were used without further purification. Doubly distilled water passed through a MilliQ apparatus was utilized.

### 2.1. Synthesis of Both Pristine and Hybrid GO-Based Compounds

Graphene oxide (GO) was prepared by adopting a modified Hummers’ method, already reported elsewhere [27,35]. For the solid solution Sn_x_Ti_1−x_O_2_/GO materials, different tin(IV) chloride pentahydrate (SnCl_4_ × 5H_2_O, by Sigma-Adrich, Darmstadt, Germany, 98%) contents were dissolved in 3.0 g L^−1^ of a 2-propanol-based (by Sigma-Aldrich, ≥95.5%) GO suspension to produce both Sn/(Sn + Ti) precursor molar ratios equal to 0.21, 0.35, 0.44, 0.55 and 0.71, and Sn + Ti salt precursors-to-GO weight ratios always of 32:1. The mixture was stirred (at 300 rpm) for 1.5 h at 50 °C and, then, the required amount of titanium (IV) isopropoxide (Ti(OCH(CH_3_)_2_)_4_, TTIP, by Sigma-Aldrich, 97%) was added to the previous mixture, accordingly. Then, the system was left under stirring for another 1.5 h. Subsequently, 2 M sodium hydroxide (NaOH, by Sigma-Aldrich, ACS reagent) was added dropwise into the flask in a stoichiometric amount, related to the sum of tin- and titanium-precursors moles, and the mixture was continuously stirred for other 3 h. The resulting product was centrifuged (at 8000 rpm) several times with MilliQ water, until the pH became neutral. Afterwards, it was dried in an oven at 60 °C. Finally, a calcination step at 400 °C, under oxygen flux (6 h, 9 NL h^−1^) was carried out to form a whitish precipitate.

Notably, the chemical composition of the system was varied over a full range from 100 mol% of SnO_2_ (namely, 32:1 SnO_2_/GO) to 100 mol% of TiO_2_ (namely, 32:1 TiO_2_/GO). These oxide-based composites were labelled as: 32:1 Sn_x_Ti_1−x_O_2_/GO, where x = 0.21, 0.35, 0.44, 0.55 and 0.71.

For the sake of comparison, pure SnO_2_ and TiO_2_ powders were prepared through the same synthetic route and bare rutile TiO_2_ (purchased from Sigma-Aldrich, 99.5% trace metals basis) was also taken into account.

### 2.2. Powders Physico-Chemical Characterizations

X-Ray Powder Diffraction (XRPD) analyses were performed on a Philips PW 3710 Bragg-Brentano goniometer equipped with a scintillation counter, 1° divergence slit, 0.2 mm receiving slit and 0.04° Soller slit systems. We used graphite-monochromated Cu K_α_ radiation (Cu K_α1_ *λ* = 1.54056 Å, K_α2_ *λ* = 1.54433 Å) at 40 kV × 40 mA nominal X-rays power. Diffraction patterns were collected between 10° and 70° with a step size of 0.1°. A microcrystalline Si-powder sample was employed as a reference to correct instrumental line broadening effects.

Raman spectra were recorded on pure samples using a Bruker Vertex 70 spectrometer (Billerica, MA, USA), equipped with the RAMII accessory and Ge detector, by exciting samples with Nd:YAG laser source (1064 nm), with resolution of 4 cm^−1^.

Specific surface area and porosity distribution were determined from N_2_ adsorption/desorption isotherms at 77 K using a Micromeritics Tristar II 3020 (Norcross, GA, USA) apparatus and the instrumental software (Version 1.03) by applying Brunauer-Emmett-Teller (BET) and Barrett-Joyner-Halenda analyses, respectively. Before measurements, sample powders were pretreated at T = 150 °C (4 h under N_2_ flux) to remove adsorbed species.

Scanning Electron Microscopy (SEM)/Energy Dispersive X-ray spectroscopy (EDX) were carried out using a SEM Hitachi TM-1000 coupled with Hitachi ED3000 spectrophotometer (Krefeld, Germany).

Transmission Electron Microscope (TEM) analyses were performed on a JEOL JEM 3010UHR microscope operating at 300 kV and equipped with a LaB6 single crystal filament and an energy dispersive X-ray (EDX) detector (Oxford INCA Energy TEM 200, Oxford, UK). Sample powders were dry deposited on 200 mesh Cu “holey” carbon grids before the analyses.

ATR-FTIR spectra were recorded using a Nicolet 380 spectrophotometer (Thermo Electron Corporation, Waltham, MA, USA) between 4000 and 1400 cm^−1^.

X-ray Photoelectron Spectroscopy (XPS) (Thermo Scientific, Waltham, MA, USA) analysis was carried out by means of an M-probe apparatus (Surface Science Instrument), using a monochromatic Al K_α_ radiation source (1486.6 eV). The XPS binding energy scale was charge corrected using the standard calibration, fixing the C 1s peak at 284.6 eV.

To evaluate powders’ optical band gaps by Kubelka-Munk elaboration, Diffuse Reflectance Spectra (DRS) were measured on a UV/Vis spectrophotometer Shimadzu UV-2600 spectrophotometer (Kyoto, Japan) equipped with an integrating sphere; a “total white” BaSO_4_ powder was used as reference.

### 2.3. Deposition on Au-Interdigitated Electrodes (Au-IDEs) and VOCs Sensing Tests

Powders were deposited on glass substrates topped with interdigitated Au electrodes (Au-IDEs) by a simple hot-spray method reported in our previous works [27,35]. Therefore, the tested IDEs were prepared by adopting both pristine and solid solution metal oxides.

Acetone and toluene species were chosen as target analytes for the VOCs sensing measurements. The tests were performed using a homemade stainless-steel cell. The IDE-coated sensors were placed above a hotplate connected to a PID temperature controller. The upper part of the cell was closed with a quartz window for UV/Vis irradiation of the investigated sensors. The tests were made under flowing 20 vol.%O_2_/N_2_ gas mixture (0.5 L min^−1^ flow rate; Alphagaz^TM^ Air Liquid, 99.999 mol% purity, humidity traces) regulated by mass flow controllers (Bronkhorst, The Netherlands). The desired amount of target gases up to 4 ppm in 20 vol.%O_2_/N_2_ (0.5 L min^−1^ flow rate) was introduced into the cell by means of a four-way switching valve. The analyte concentration was varied by dilution of a proper flow of a starting 500 ppm acetone or toluene in N_2_ gas mixture (Sapio S.r.l., relative uncertainty 2.0%, humidity traces) keeping constant the total flow rate of 0.5 L min^−1^. The sensing tests under UV light irradiation were made at operating temperature equal or below 250 °C using an iron halide mercury arc lamp (Jelosil HG500, 500 W) as an irradiation source, emitting in the 350–450 nm range with an incident power density of 30 mW cm^−2^. For the gas sensing tests, two gold probes were separately placed on top of the powders covered IDEs, and the dynamic response was recorded by an electrochemical workstation (Autolab PGStat30, Ecochemie The Netherlands, potentiostat/galvanostat controlled by NOVA 2.0 software), applying a bias of +1.0 V. The sensor response is reported as: (R_air_/R_analyte_) − 1, where R_air_ is the film resistance in air and R_analyte_ is the film resistance at a given concentration of the target gas [42]. As reported in our previous works [27,35], both sensors’ response and recovery times have been evaluated considering the 90% of the final response.

## 3. Results and Discussion

### 3.1. Nanostructured Sn_x_Ti_1−x_O_2_/GO Solid Solutions: Composition and Physico-Chemical Properties

Nanostructured Sn_x_Ti_1−x_O_2_/GO solid solutions were finely investigated to deeply understand the correlation between their physico-chemical properties and the sensing performances.

**Figure 1:**
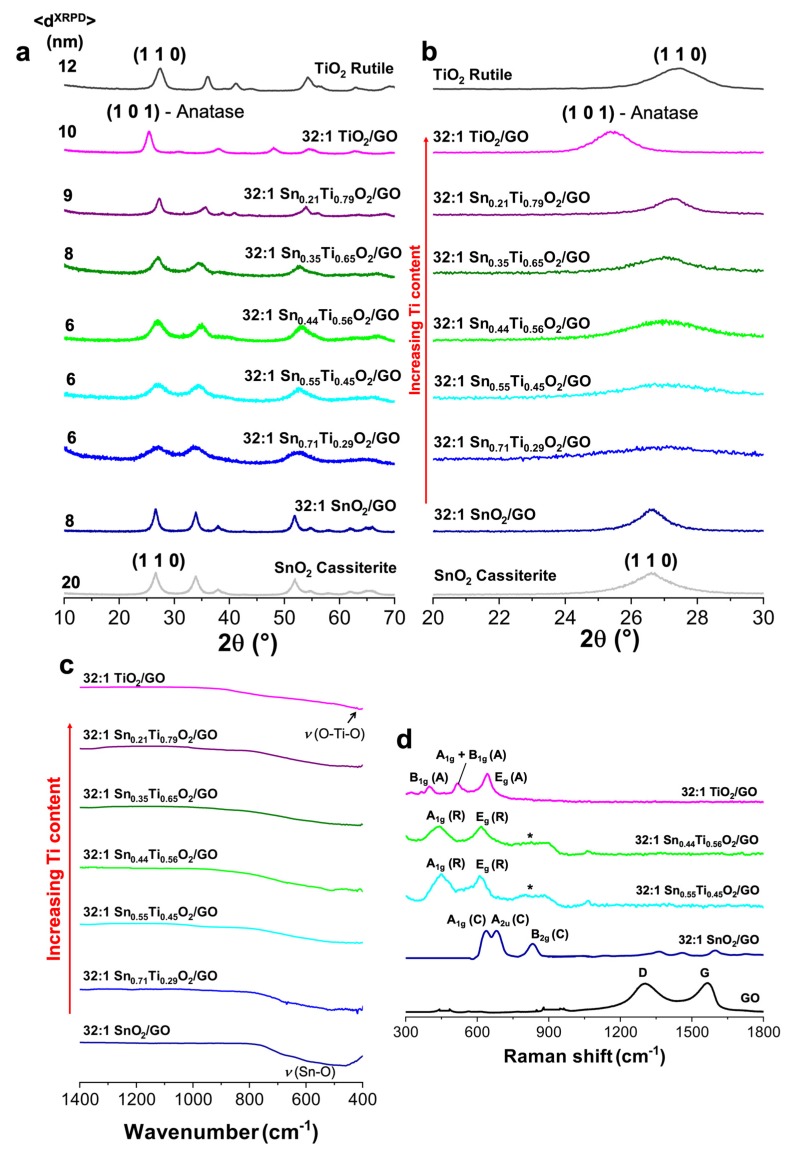
(**a**) Comparison of X-ray diffraction lines relative to pure cassiterite SnO_2_, rutile TiO_2_, 32:1 SnO_2_/GO, 32:1 TiO_2_/GO and all the Sn_x_Ti_1−x_O_2_/GO solid solutions by increasing the titanium content. (**b**) Magnification of the X-Ray Powder Diffraction (XRPD) patterns to investigate the shift of the more intense peak from 26.6° of pure cassiterite SnO_2_ [40,45] to 27.4° of pure rutile TiO_2_ [40]. (**c**) FTIR spectra of the tested samples, (**d**) Raman data of pure GO, 32:1 SnO_2_/GO, 32:1 TiO_2_/GO, 32:1 Sn_0.55_Ti_0.45_O_2_/GO, 32:1 Sn_0.35_Ti_0.65_O_2_/GO samples (A = anatase, R = rutile and C = cassiterite). * = Traces of SnO_2_ cassiterite.

Wide-angle XRPD analyses were performed to determine the crystal structure and to corroborate the formation of Sn/Ti solid solutions. Figure 1a reports the XRPD patterns of the as-synthesized nanostructured metal oxides with a controlled Sn/Ti ratio. Notably no GO traces, whose 100% intensity peak is located at 2θ = 12° [43], were observable in all the diffraction patterns. Moreover, the crystallinity degree of 32:1 Sn_x_Ti_1−x_O_2_/GO composites seems to slightly increase with increased titanium content (i.e., sharper peaks starting from 32:1 Sn_0.35_Ti_0.65_O_2_/GO sample), having estimated grain sizes from about 6 to 10 nm. Conversely, a drop in crystallinity was observed passing from bare SnO_2_ (ca. 20 nm) to 32:1 SnO_2_/GO (8 nm) and 32:1 Sn_0.71_Ti_0.29_O_2_/GO, until x = 0.44 (6 nm), above which a smooth rise was noticed again. Besides, as clearly visible in Figure 1b, all the 32:1 Sn_x_Ti_1−x_O_2_/GO exhibit no phase segregation: the main cassiterite SnO_2_ peak (1 1 0; JCPDS 14-1445 [40]) slightly shifts from 26.6° (of pure SnO_2_ and 32:1 SnO_2_/GO) to 27.4° typical of rutile TiO_2_ (JCPDS n. 21-1276 [40]), as the Ti content increases. Interestingly, in the investigated solid solutions, the anatase polymorph (JCPDS n. 21-1272 [40]) was not observed, except in 32:1 TiO_2_/GO sample, being cassiterite and rutile-TiO_2_ isostructural phases (tetragonal cell [44]). These results show that Ti atoms could substitute Sn ones, maintaining the structural configuration more similar to cassiterite (rutile-type) polymorph, without drastic cell distortions.

Furthermore, the effect of Ti-content was also investigated by FTIR and Raman spectroscopies (Figure 1c,d). Notably, Figure 1c reports the non-linear, very rapid transition from SnO_2_ (~500 cm^−1^, ascribable to Sn–O stretching [46]) to TiO_2_ (~470 cm^−1^, ascribable to O–Ti–O stretching [38]) infrared modes, which is already almost accomplished in the 32:1 Sn_0.71_Ti_0.29_O_2_/GO sample. According to Tricoli et al. [37], even 9% of Ti-doping into SnO_2_ matrix can affect the ionic transport in the crystal, the molecular bond length and, thus, the surface adsorption properties/sensing performances. Raman analyses corroborate the previous XRPD and FTIR results. Going into detail, the main transition from the anatase polymorph, typical of 32:1 TiO_2_/GO, to the rutile one, present in the solid solution oxides, occurs by decreasing the Ti content (Figure 1d). Specifically, in the case of 32:1 TiO_2_/GO the four Raman modes peculiar of anatase TiO_2_ are evident: the E_g_ at 641 cm^−1^, the A_1g_ and B_1g_ at 524 cm^−1^ and the B_1g_ at 398 cm^−1^ [37]. Contrarily, as the titanium content decreases, the E_g_ and A_1g_ rutile peaks of TiO_2_ become evident at 612 and 445 cm^−1^, respectively [37]. This abrupt transition from anatase to rutile is consistent with XRPD results (Figure 1a,b). Moreover, a further reduction of the Ti amount gradually shifts the TiO_2_ rutile Raman modes to the SnO_2_ cassiterite ones, clearly visible in the 32:1 SnO_2_/GO spectrum (see Figure 1d, A_1g_ at 624 cm^−1^, A_2u_ at 640 cm^−1^ and B_2g_ at 766 cm^−1^).

The specific surface area (*S*_BET_) of the investigated samples rises with the increasing of the Ti content (Table 1), from 60 m^2^ g^−1^ of 32:1 SnO_2_/GO up to of 177 m^2^ g^−1^ of 32:1 Sn_0.44_Ti_0.56_O_2_/GO, followed by a drastic decrease at higher titanium amounts.

This trend is in agreement with the increasing crystallinity observed by XRPD and due to an increasing particle size attained at high Ti content, i.e., at the phase transition from a cassiterite-like to a rutile-like crystal structure [47,48]. Interestingly, the same trend was observed for the total pores volume (see Table 1, 3rd column and Figure S1c). Figure S1c clearly evidences that the majority of pores present in the solid solutions are smaller than 5 nm, whereas 32:1 SnO_2_/GO and 32:1 TiO_2_/GO show a different distribution, having pores slightly larger (the 90% are below 10 nm). In particular, Figure S1b displays that by increasing the titanium amount, pores tend to get smaller, showing the distribution peak at around 3–4 nm. However, the 32:1 Sn_0.21_Ti_0.79_O_2_/GO compound represents an exception, since it has a very small total pore volume, and the corresponding Gaussian curve has a very broad band centered at around 12 nm. In any case, either 32:1 SnO_2_/GO or 32:1 TiO_2_/GO possess pores with a slightly higher average dimensions, of ca. 5–6 nm. Furthermore, BET isotherms (Figure S1a) unveil the presence of bottle-necked pores [49], with a typical H2-type hysteresis loop for pure TiO_2_, SnO_2_ and 32:1 TiO_2_/GO. However, 32:1 Sn_x_Ti_1−x_O_2_/GO materials, along with 32:1 SnO_2_/GO, are characterized by a hysteresis loop shape between that of pure SnO_2_ or TiO_2_ and the one of GO [35]. Therefore, some of these powders possess bottle neck-shaped pores (typical of SnO_2_), others slit-shaped ones (typical of GO).

**Figure 2:**
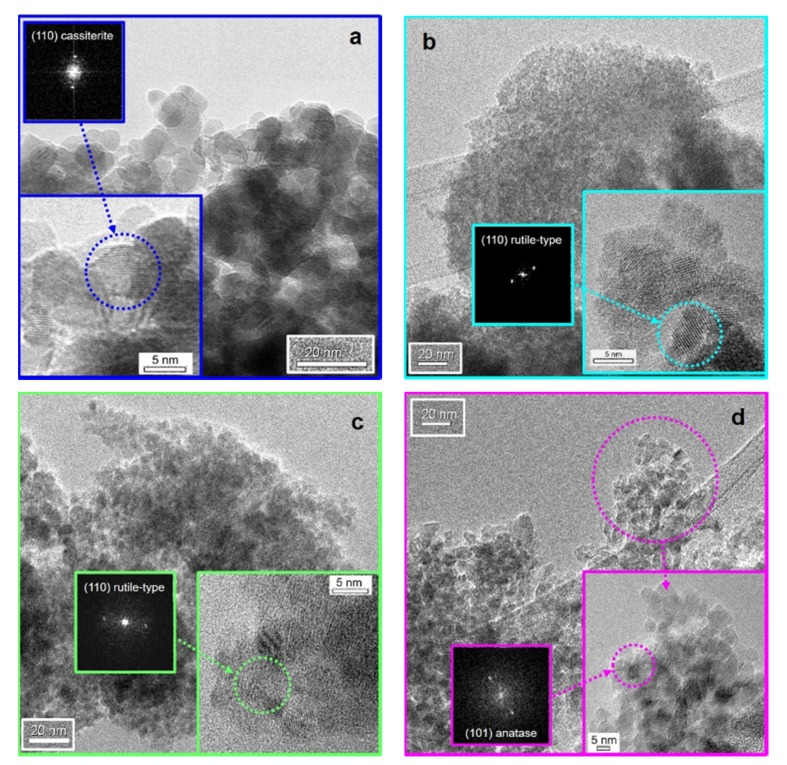
TEM images of (**a**) 32:1 SnO_2_/GO, (**b**) 32:1 Sn_0.55_Ti_0.45_O_2_/GO, (**c**) 32:1 Sn_0.44_Ti_0.56_O_2_/GO and (**d**) 32:1 TiO_2_/GO, as representative samples (insets: relative magnifications together with the relative Selected Area Electron Diffraction, SAED, patterns).

Figure 2 and Figure S2 refer to both morphological (i.e., surface texture) and external habits of some of the as-prepared powders, by both TEM and SEM measurements, respectively. Specifically, from SEM micrographs (Figure S2), it is possible to observe the presence of micrometric spherical aggregates, even of about 200 µm, especially for low Ti-content. Conversely, by increasing the titanium amount, these agglomerates tend to become even smaller (<100 µm), exhibiting a characteristic sponge-like morphology (Figure S2f), that is much more similar to the 32:1 TiO_2_/GO material (Figure S2g).

In addition, the EDX analyses (Figure S2) confirm the starting amount of Sn and Ti in the solid solutions. Parallel to this, TEM images reveal the presence of nanometric crystalline particles in either cassiterite 32:1 SnO_2_/GO (Figure 2a) or anatase TiO_2_/GO (Figure 2d) phases. In contrast, the solid solutions 32:1 Sn_x_Ti_1−x_O_2_/GO exhibit a lower general crystallinity, as a further corroboration of the XRPD results. In particular, for these mixed samples, it seems that SnO_2_ behaved as seeds for the further growth of the rutile titania nanoparticles. Moreover, as already discussed for the XRPD outputs, the crystallinity degree seems to slightly rise with increasing titanium content, i.e., from the 32:1 Sn_0.35_Ti_0.65_O_2_/GO sample to the 32:1 TiO_2_/GO one. This feature is further confirmed by the investigation carried out at high resolution (see the inset to Figure 2b), relative to 32:1 Sn_0.55_Ti_0.45_O_2_/GO: only starting from this ratio between Ti and Sn on, it is possible to single out frequent crystalline fringe patterns (ascribable, if inspected in detail, to the (1 1 0) crystal planes of rutile-type structure with d(hkl) = 0.332 nm: see the electron diffraction in the inset to Figure 2b [ICDD card n. 43-1002]). For smaller ratios between Ti and Sn, a more amorphous nature of the materials is evidenced, as clearly observable in Figure 2c, for which only very seldom fringe patterns typical of same rutile-type structure can be singled out and confirmed by the electron diffraction patterns (see the inset of Figure 2c).

**Figure 3:**
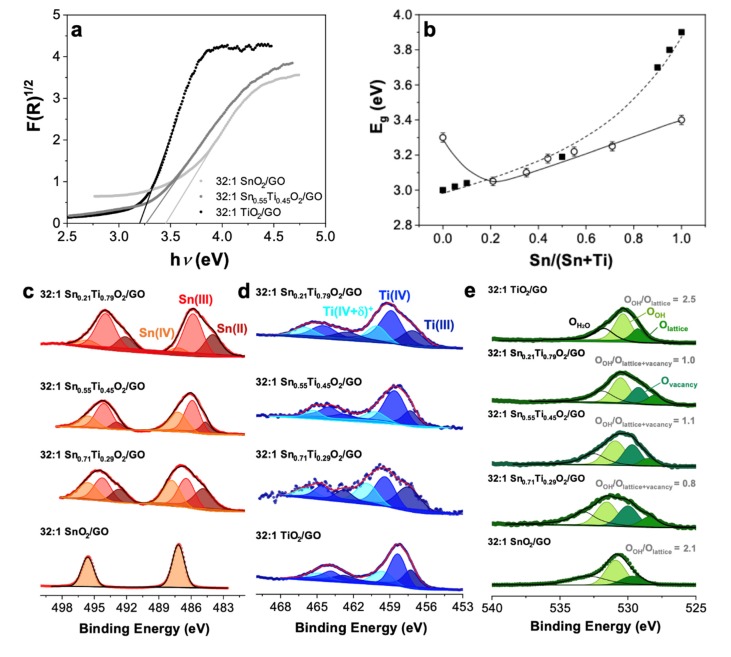
(**a**) Kubelka–Munk elaboration of the DRS spectra relative to 32:1 SnO_2_/GO, 32:1 Sn_0.55_Ti_0.45_O_2_/GO and 32:1 TiO_2_/GO. (**b**) Comparison between band gap (E_g_) values obtained herein (circles) and the ones redrawn from literature (black squares) [39] as a function of the Sn/(Sn + Ti) ratio. XP spectra of (**c**) Sn 3d, (**d**) Ti 2p and (**e**) O 1s regions relative to three 32:1 Sn_x_Ti_1−x_O_2_/GO (with x = 0.71, 0.55 and 0.21) as representative samples. 32:1 SnO_2_/GO and TiO_2_/GO have been reported for comparison.

As far as the optical properties are concerned, DRS analyses were performed and, subsequently, elaborated by Kubelka–Munk equation (Figure 3a). Notably, as the amount of titanium ions into tin dioxide lattice increases, the band gap value tends to decrease (Figure 3b). Indeed, E_g_ shifts from 3.40 eV of 32:1 SnO_2_/GO to 3.05 eV of 32:1 Sn_0.21_Ti_0.79_O_2_/GO, reaching a value much that is much more similar to the pure TiO_2_ one (i.e., 2.90–3.00 eV). These results are further confirmed by the literature, i.e., the same trend has been already reported by Zakrzewska et al. [39] for SnO_2_–TiO_2_ materials (Figure 3b), pointing out the systematic change of the solid solutions band gap with the increasing of titanium content.

Furthermore, surface properties by XPS measurements were finely investigated to give insights into powder composition by varying the Ti content. Hence, three different Sn/(Sn + Ti) ratios, i.e., the lowest 0.21, an intermediate 0.55 and the highest 0.71 ones, were thoroughly studied.

Particularly, the Sn 3d_5/2_ region (Figure 3c) shows the presence of a peak at around 486.9 eV, typical of Sn(IV) species [44], in all the composites samples. However, while 32:1 SnO_2_/GO signal can be fitted with only this peak, the solid solutions interestingly possess broader spectra, which underline the presence of other bands. Furthermore, with the addition of titanium ions, XP spectra tend to smoothly shift towards lower binding energies and two other peaks (at ca. 484.8 and 486.3 eV) appear, which can be ascribed to Sn(III) and Sn(II) species, respectively [44]; in contrast, the peak relative to Sn(IV) slightly shifts to higher values (around 487.9 eV). Therefore, these spectral changes prove that the Sn atoms are gradually substituted by Ti cations, resulting in the formation of reduced tin ions, probably due to oxygen vacancies [44]. This observation is further supported by the area of these peaks (see Table S1): by increasing the Ti content, lower Sn valence states increase. Indeed, as observable from the ratios reported in Table S1, the Sn(II) and, above all, the Sn(III) peaks show an increased area to the detriment of the Sn(IV) peak area. Besides, focusing on the Ti 2p_1/2_ region (Figure 3d), a confirmation of the previous outcomes was obtained. Actually, for all the three solid solutions, the peaks relative to defective Ti species [50], Ti(III) (at ca. 457.1 eV) and Ti(IV + δ)^+^ (at ca. 458.6 eV, [51]) are observable. In particular, with the increasing of titanium atoms, the area of Ti(IV + δ)^+^ rises by about 30% due to the presence of Sn(III) and Sn(II) species (Table S1) and, hence, to the much higher lattice defectivity. Precisely, this defectivity was verified by focusing on the O 1s region: only the 32:1 Sn_x_Ti_1−x_O_2_/GO materials show the peak at ca. 530.2 eV that is reported to be ascribable to a high binding energy component (HBEC) developed with the increasing loss of oxygen or creation of oxygen vacancies [52,53]. Besides, the other peaks are respectively correlated to: (i) oxygen bound to the metal ions (Ti [51,54] or Sn [48]) in the lattice (at ca. 528.6 eV), (ii) a low binding energy component (LBEC) due to adsorption of OH^−^ on the surface (at ca. 531.1 eV [51,52,55]); and (iii) water adsorption (at B.E. equal or higher than 532.0 eV) [51,56].

### 3.2. VOCs (Toluene and Acetone) Sensing

As widely reported in the literature [13,15,16,57], both oxide-based compounds possess optimal features as sensing materials for very small and high-polar molecules, such as gaseous ethanol. In this research, instead, the solid solution oxides have been tested towards a bigger and non-polar analyte, toluene. Furthermore, the adopted scalable air-spraying method, already reported in our previous works [27,35], led to very homogeneous micrometric films, having an average thickness of about 1.5–3.0 µm for all the tested materials. These values are fully in agreement with the best performing micrometers films already described in the literature for gas sensing applications [58,59].

Specifically, gas sensing measurements were conducted first at high temperatures (350 °C, without UV light), and secondly at decreased T by exploiting the synergistic effect between n-type semiconductors and p-type GO [27,35]. For comparison, tests were also performed using pure SnO_2_ and TiO_2_, synthesized through the same route: in both cases, no reproducible and good responses were obtained.

**Figure 4:**
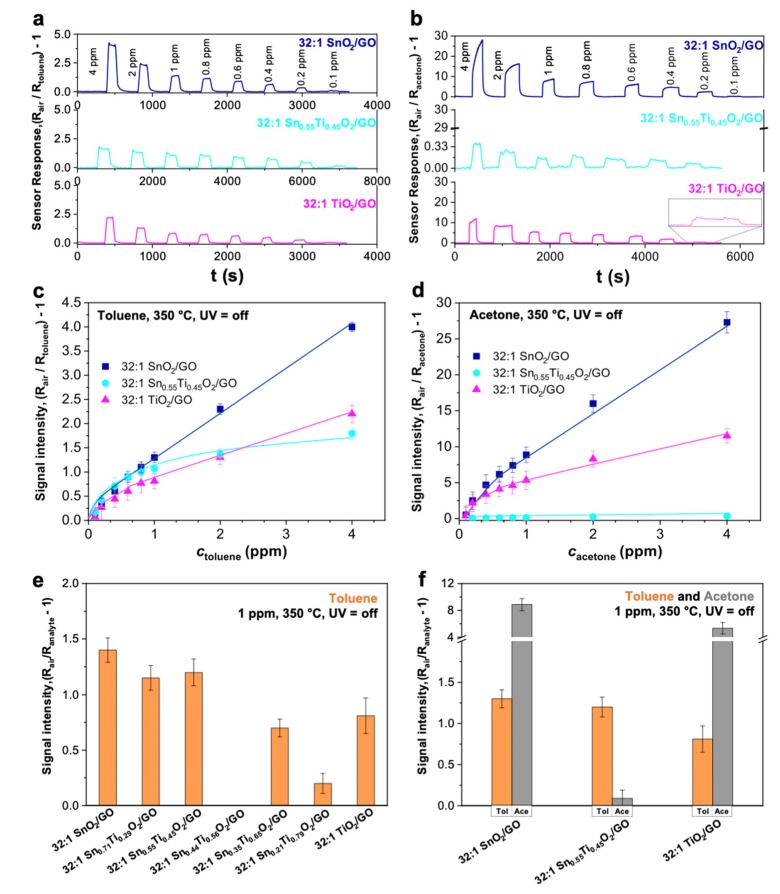
32:1 SnO_2_/GO (blue line), 32:1 Sn_0.55_Ti_0.45_O_2_/GO (cyan line) and 32:1 TiO_2_/GO (fuchsia line) sensor signals towards (**a**) toluene and (**b**) acetone molecules. Sensor response intensities towards (**c**) toluene and (**d**) acetone species. (**e**) Histograms displaying a comparison of the response intensity obtained with the solid solutions towards 1 ppm of toluene analyte. (**f**) Comparison of the intensities at 1 ppm of toluene (orange) and acetone (grey) obtained with the most promising 32:1 SnO_2_/GO, 32:1 TiO_2_/GO and 32:1 Sn_0.55_Ti_0.45_O_2_/GO samples. Tests were carried out in simulated air (20% O_2_–80% N_2_) at 350 °C, without UV light.

Figure 4a and Figure S3 show the responses obtained with 32:1 SnO_2_/GO, 32:1 TiO_2_/GO and the 32:1 Sn_x_Ti_1−x_O_2_/GO materials towards different toluene concentrations (from 4 to ppb-level), at 350 °C. Remarkably, by increasing the titanium amount, the sensors response worsens (see Figure S3e relative to 32:1 Sn_0.21_Ti_0.79_O_2_/GO), even resulting in absence of any change in conductivity for the 32:1 Sn_0.44_Ti_0.56_O_2_/GO sample (4th histogram in Figure 4e). The reason for this behavior has been already unraveled by Tricoli et al. [37], who investigated the electrical and chemical properties of SnO_2_–TiO_2_ solid solutions. Specifically, they observed that the resistivity of these materials increases up to 7 orders of magnitude by increasing the Ti-content from 0 to 80%. Thus, this strong increase in resistivity may indicate the dominant role of the surface states in determining the solid solution conductivity. Moreover, in our particular case, notwithstanding that the 32:1 Sn_0.44_Ti_0.56_O_2_/GO compound possesses the highest surface area (177 m^2^ g^−1^; Table 1, 2nd column), it is also one of the least crystalline materials, showing very large peaks and an amorphous phase, as observed from XRPD analysis and corroborated by TEM images. Zakrzewska et al. [39] already described a similar performance trend, evidencing a minimum of H_2_ sensing for Sn/(Sn + Ti) of 0.5. Therefore, samples with a slightly higher Sn content seem to be preferable for VOC sensing. Herein, the best performing samples, in terms of either sensitivity (down to 100 ppb of toluene, Figure S3) or signal intensity (see histograms for 1 ppm-response, in Figure 4e) seem to be 32:1 SnO_2_/GO, 32:1 TiO_2_/GO and 32:1 Sn_0.55_Ti_0.45_O_2_/GO. Indeed, both solid solutions having x = 0.21 and 0.55 showed greater response intensities; however, only with 32:1 Sn_0.55_Ti_0.45_O_2_/GO was the Limit of Detection (LOD) reached 100 ppb (Figure S3). Besides, by comparing the curves displayed in Figure 4c, the solution of 32:1 Sn_0.55_Ti_0.45_O_2_/GO seems to reach a plateau region at high toluene concentrations, contrarily to both the 32:1 SnO_2_/GO and the 32:1 TiO_2_/GO behavior. This may be connected to the different pore size distributions, since only the solid solutions have a higher percentage of pores smaller than 5 nm (Figure S1c). Hence, this feature could have played a significant role in the response recovery, i.e., the higher the percentage of very small pores, the more difficult the desorption of the sensing products and, thus, the signal recovery (as demonstrated by the greater recovery times in Table S2). Nevertheless, the three most promising chemoresistors have shown very sharp signals, indicating short response/recovery times (of about 20–35 s and 30–60 s, respectively; see Table S2a).

Starting from all the previous findings, the optimal samples were tested towards acetone analyte, to eventually elucidate their potential selectivity towards a specific biomarker. Thus, Figure 4f reports the comparison of the 1 ppm signal intensity for all the three compounds, always at high operating temperatures of 350 °C. Moreover, the relative sensor responses are reported in Figure 4b. Two main observations can be made: regarding the sensitivity and the signal intensities, 32:1 SnO_2_/GO and 32:1 TiO_2_/GO (Figure 4a–d) seem to better sense acetone with respect to toluene gaseous molecules. Remarkably, in Figure 4b, it is clearly visible that, while 32:1 SnO_2_/GO (blue line) and 32:1 TiO_2_/GO (fuchsia line) have a very similar behavior of the intensity signal with the variation of the VOCs concentrations, the 32:1 Sn_0.55_Ti_0.45_O_2_/GO solid solution resulted in a dramatic decrease in the response (cyan line). Indeed, both 32:1 SnO_2_/GO and 32:1 TiO_2_/GO display an almost six-fold signal intensity relative to the ones achieved in the presence of toluene analyte (at the same concentration and experimental conditions; see Figure 4c,d). Secondly, the 32:1 Sn_0.55_Ti_0.45_O_2_/GO solid solution seems to possess much more selectivity towards toluene than acetone, as noticeable in Figure 4f, notwithstanding the low signal intensities. Interestingly, the sensitivity also appears to be affected as, in the case of acetone, 200 ppb was the lowest detectable concentration (instead of 100 ppb for toluene; Figure 4a,b). Indeed, as already unveiled by Tricoli et al. [37], a low titanium content limits the cross-sensitivity to relative humidity by reducing the number of rooted and terminal OH surface species with respect to both pure metal oxides. A further corroboration of this assertion was attained by investigating the high-resolution XPS O 1s region of 32:1 SnO_2_/GO, 32:1 TiO_2_/GO and solid solutions (see Figure 3e). By computing the ratio between the area relative to the adsorbed hydroxyl groups and those ascribable to oxygen lattice and vacancies, it is clearly visible that the solid solutions are less hydrophilic than 32:1 SnO_2_/GO and 32:1 TiO_2_/GO materials. Hence, herein, a lower hydrophilicity of the solid solutions surface may have led to a scarcer affinity to polar analytes, such as acetone, thus favoring the sensing of bigger and non-polar toluene molecules. Moreover, as largely discussed for XRPD results, 32:1 Sn_x_Ti_1−x_O_2_/GO compounds are mainly constituted by the rutile polymorph, which is already shown to have a lower amount of surface hydroxyl groups than the anatase phase, as conversely 32:1 TiO_2_/GO shows [60,61,62]. As such, together with the so far reported higher catalytic activity of the rutile TiO_2_ towards toluene species [63], the greater selectivity to low/non-polar molecules of the solid solutions may be justified. Finally, response and recovery times obtained with acetone are well comparable to those reached towards toluene, and all below 70 s (see Table S2b). Remarkably, the 32:1 TiO_2_/GO material shows very short response and recovery times of 20 and 30 s respectively, which are fully consistent with some of the best performing MOS-based chemoresistors [1,10,22,33,64].

**Figure 5:**
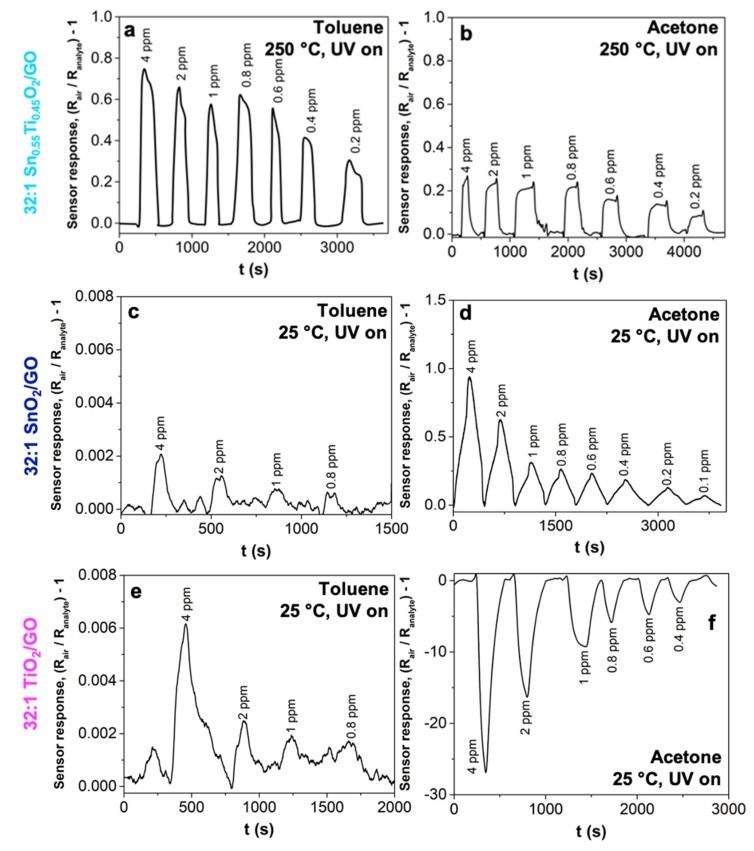
(**a**) Toluene and (**b**) acetone sensing by 32:1 Sn_0.55_Ti_0.45_O_2_/GO solid solution, at 250 °C with UV light. Sensor responses obtained towards (**c**,**e**) toluene and (**d**,**f**) acetone for 32:1 SnO_2_/GO and 32:1 TiO_2_/GO, at RT with UV irradiation. All the measurements were carried out in simulated air (20% O_2_–80% N_2_). OT = Operating Temperature.

Additionally, our aim was to reduce the operating temperature. Thus, Figure 5 shows a comparison between toluene and acetone sensing by the adopted 32:1 Sn_0.55_Ti_0.45_O_2_/GO solid solution, at 250 °C (with the aid of the UV light). Notably, with this sample, it was not possible to further reduce the temperature, even exploiting the UV irradiation. For both gases, 200 ppb were detected (Figure 5a,b) and the highest selectivity towards bigger and non-polar analytes, as toluene, has been preserved. In contrast, with the 32:1 SnO_2_/GO and 32:1 TiO_2_/GO (Figure 5c–f) composites, a signal was obtained even at RT, UV light aided. Specifically, both samples conserve the highest sensitivity and goodness of sensor signal towards acetone molecules, rather than toluene gas. However, interestingly, the 32:1 TiO_2_/GO sample exhibits a “reversed” behavior in the case of small and polar molecules, such as acetone (Figure 5f), maybe caused by the interaction between the moisture adsorbed on the sensing material highly-hydrophilic surface [60] and the VOC molecules, and this is lost when the operating temperature is above the water condensation point, as reported by Tricoli et al. [65].

Overall, the tailoring of titanium ions into tin dioxide matrix alongside with the integration of graphene oxide into the metal oxides could be exploited for the engineering of materials with higher sensing performances, especially in terms of selectivity to different VOC species.

## 4. Conclusions

In this study, 32:1 Sn_x_Ti_1−x_O_2_/GO materials were synthesized via a simple hydrothermal route, varying the relative Sn/Ti ratios. All these materials have been thoroughly investigated from structural, morphological, surface and optical points of view, showcasing tailored features depending on the titanium amount. Furthermore, the toluene and acetone gas sensing performances of the as-prepared sensors were systematically investigated and compared with 32:1 SnO_2_/GO and 32:1 TiO_2_/GO nanocomposites. Specifically, the latter exhibited a greater selectivity towards acetone analyte, also at room temperature, down to hundreds of ppb. Contrarily, solid solutions possessing a higher content of tin with respect to titanium, as 32:1 Sn_0.55_Ti_0.45_O_2_/GO, evidenced higher selectivity towards bigger and non-polar molecules (such as toluene) at 350 °C, rather than acetone. A lower hydrophilicity surface texture, together with the simultaneous presence of smaller pores (3–4 nm), could explain the sensing features towards non-polar molecules. However, by exploiting either the presence of graphene oxide embedded in the MOS-based solid solutions or the UV light, a reduction of the operating temperature was successfully attained. Indeed, a significant and reliable signal was recorded down to 250 °C, interestingly keeping a much greater selectivity towards toluene analyte. In contrast, at RT, 32:1 TiO_2_/GO showed an enhanced opposite change of conductivity to acetone, probably due to its highly hydrophilic surface composition, which causes a greater adsorption of moisture at RT, thus competing with the target VOC for the adsorption sites. Therefore, an opposite response to that expected for n-type semiconductors with reducing gases may occur. Hence, we believe that the feasibility of tuning the chemical selectivity, by engineering the relative amount of SnO_2_ and TiO_2_, grown on graphene oxide, can provide guidelines for the engineering of the next room-temperature chemoresistors for the selective detection of VOCs.

## Figures and Tables

**Table 1:** Surface area (*S*_BET_) and total pore volume (V_tot. pores_) by BET-BJH analysis, together with the relative standard deviations directly given by the instrumental software.

Sample	*S*_BET_ (m^2^ g^−1^)	V_tot. pores_ (cm^3^ g^−1^) ^♣^
Graphite	11 ± 1	0.030
GO	45 ± 1	0.034
SnO_2_ Cassiterite	67 ± 1	0.210
32:1 SnO_2_/GO	60 ± 1	0.130
32:1 Sn_0.71_Ti_0.29_O_2_/GO	65 ± 1	0.048
32:1 Sn_0.55_Ti_0.45_O_2_/GO	117 ± 2	0.119
32:1 Sn_0.44_Ti_0.56_O_2_/GO	177 ± 2	0.170
32:1 Sn_0.35_Ti_0.65_O_2_/GO	44 ± 1	0.070
32:1 Sn_0.21_Ti_0.79_O_2_/GO	34 ± 1	0.120
32:1 TiO_2_/GO	159 ± 2	0.303
TiO_2_ anatase	132 ± 2	0.250
Commercial TiO_2_ rutile	<10	0.033

**^♣^** The relative standard deviation values are around 3–4%.

## Supplementary Materials

The following are available online at https://www.mdpi.com/2079-4991/10/4/761/s1, Table S1: Binding Energies (B. E.) relative to different Sn and Ti oxidation states, and ratios between counts of each peak and the total counts, Table S2: Response (t_res_) and recovery (t_rec_) times relative to 1 ppm of (a) toluene and (b) acetone molecules, obtained at 350 °C, Figure S1: (a) Comparison of BET isotherms, (b,c) pores size distribution by BET-BJH analysis for all the solid solutions, Figure S2: SEM images alongside with the relative EDX spectra, Figure S3: Toluene sensing by (a) 32:1 SnO_2_/GO, (b–e) mixed oxides (with the exception of 32:1 Sn_0.44_Ti_0.56_O_2_/GO, since it did not show any signal), and (f) 32:1 TiO_2_/GO compounds at 350 °C.
