# Supplementary material for: Exploring SnxTi1−xO2 Solid Solutions Grown onto Graphene Oxide (GO) as Selective Toluene Gas Sensors
Source: Nanomaterials (Basel). 2020 Apr 15;10(4):761. doi: 10.3390/nano10040761 (PMC7221561; doi:10.3390/nano10040761)
Supplement: Supplementary file 1 [file nanomaterials-10-00761-s001.pdf]

nanomaterials

Supplementary Information

Exploring $Sn_xTi_{1-x}O_2$ Solid Solutions Grown onto Graphene Oxide (GO) as Selective Toluene Gas Sensors

Eleonora Pargoletti [1,2,*], Simone Verga [1], Gian Luca Chiarello [1], Mariangela Longhi [1,2], Giuseppina Cerrato [2,3], Alessia Giordana [2,3] and Giuseppe Cappelletti [1,2,*]

[1] Dipartimento di Chimica, Università degli Studi di Milano, Via Golgi 19, 20133 Milan, Italy; simone.verga@studenti.unimi.it (S.V.); gianluca.chiarello@unimi.it (G.L.C.); mariangela.longhi@unimi.it (M.L.)
[2] Consorzio Interuniversitario per la Scienza e Tecnologia dei Materiali (INSTM), Via Giusti 9, 50121 Firenze, Italy; giuseppina.cerrato@unito.it (G.C.); alessia.giordana@unito.it (A.G.)
[3] Dipartimento di Chimica & NIS, Università degli Studi di Torino, Via P. Giuria 7, 10125 Turin, Italy
* Correspondence: eleonora.pargoletti@unimi.it (E.P.); giuseppe.cappelletti@unimi.it (G.C.); Tel.: +39-02-50314228 (G.C.)

Table S1. Binding Energies (B. E.) relative to different Sn and Ti oxidation states, and ratios between counts of each peak and the total counts, for 32:1 SnO_2/GO, 32:1 $Sn_{0.71}Ti_{0.29}O_2$/GO, 32:1 $Sn_{0.55}Ti_{0.45}O_2$/GO, 32:1 $Sn_{0.21}Ti_{0.79}O_2$/GO and 32:1 TiO_2/GO representative samples.

Atom	32:1 SnO_2/GO		32:1 $Sn_{0.71}Ti_{0.29}O_2$/GO		32:1 $Sn_{0.55}Ti_{0.45}O_2$/GO		32:1 $Sn_{0.21}Ti_{0.79}O_2$/GO		32:1 TiO_2/GO	
	B. E. (eV)	Ratio	B. E. (eV)	Ratio	B. E. (eV)	Ratio	B. E. (eV)	Ratio	B. E. (eV)	Ratio
**Sn(II)**	–	–	485.3	0.38	484.9	0.12	484.2	0.33	–	–
**Sn(III)**	–	–	486.8	0.42	486.1	0.56	486.1	0.57	–	–
**Sn(IV)**	486.9	1.00	488.5	0.30	487.5	0.32	487.5	0.10	–	–
**Ti(III)**	–	–	457.5	0.40	457.3	0.36	456.4	0.32	457.3	0.33
**Ti(IV)**	–	–	459.4	0.36	458.6	0.44	458.0	0.32	458.3	0.51
**Ti(IV+δ)+**	–	–	460.9	0.24	460.2	0.20	459.1	0.36	459.6	0.16

Table S2. Response (t_{res}) and recovery (t_{rec}) times relative to 1 ppm of (**a**) toluene and (**b**) acetone molecules, obtained at 350 °C, without UV light, in simulated air (20% O_2 – 80% N_2).

(**a**)

Sensor	t_{res} (s)	t_{rec} (s)
32:1 SnO_2/GO	20	30
32:1 $Sn_{0.71}Ti_{0.29}O_2$/GO	50	55
32:1 $Sn_{0.55}Ti_{0.45}O_2$/GO	36	65
32:1 $Sn_{0.44}Ti_{0.56}O_2$/GO	–	–
32:1 $Sn_{0.35}Ti_{0.65}O_2$/GO	55	60
32:1 $Sn_{0.21}Ti_{0.79}O_2$/GO	100	50
32:1 TiO_2/GO	25	30

(**b**)

Sensor	t_{res} (s)	t_{rec} (s)
32:1 SnO_2/GO	65	70
32:1 $Sn_{0.55}Ti_{0.45}O_2$/GO	30	50
32:1 TiO_2/GO	20	30

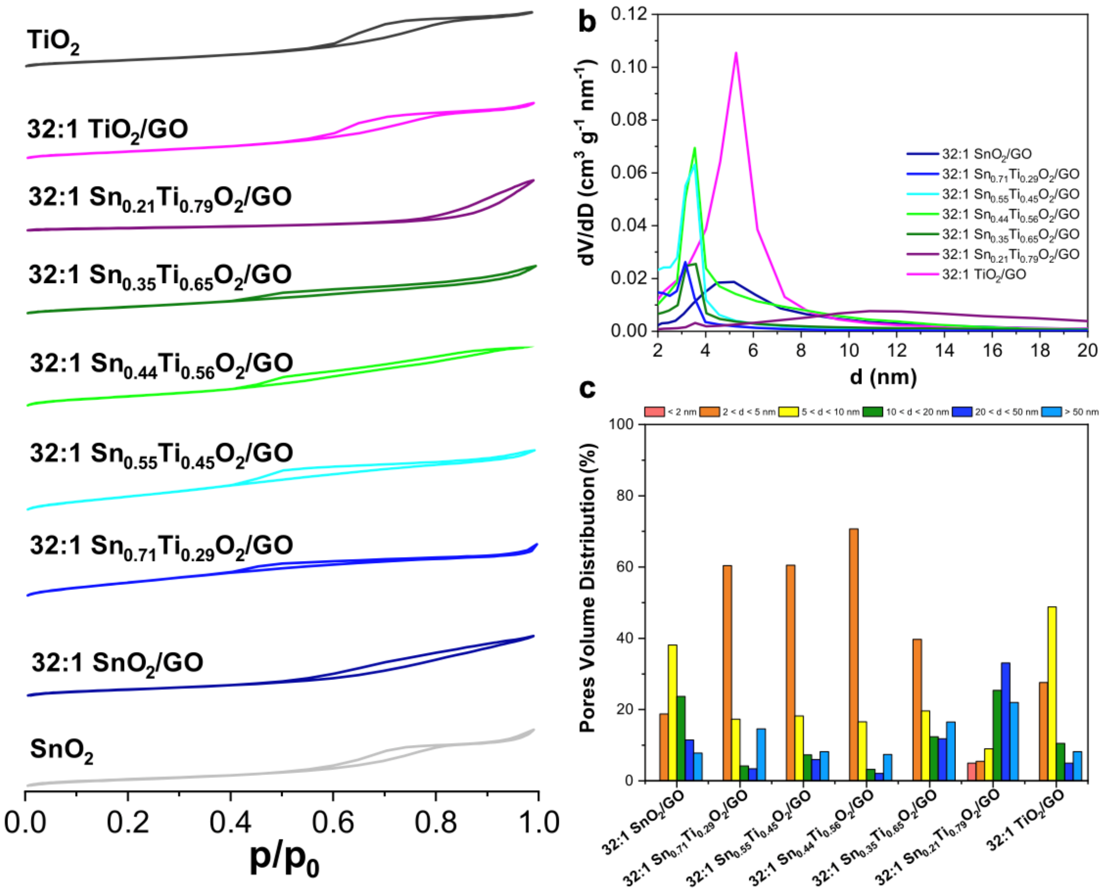

Figure S1. (**a**) Comparison of BET isotherms from pure cassiterite SnO_2, 32:1 SnO_2/GO to 32:1 TiO_2/GO, pure anatase TiO_2. (**b**,**c**) Pores size distribution by BET-BJH analysis for all the solid solutions.

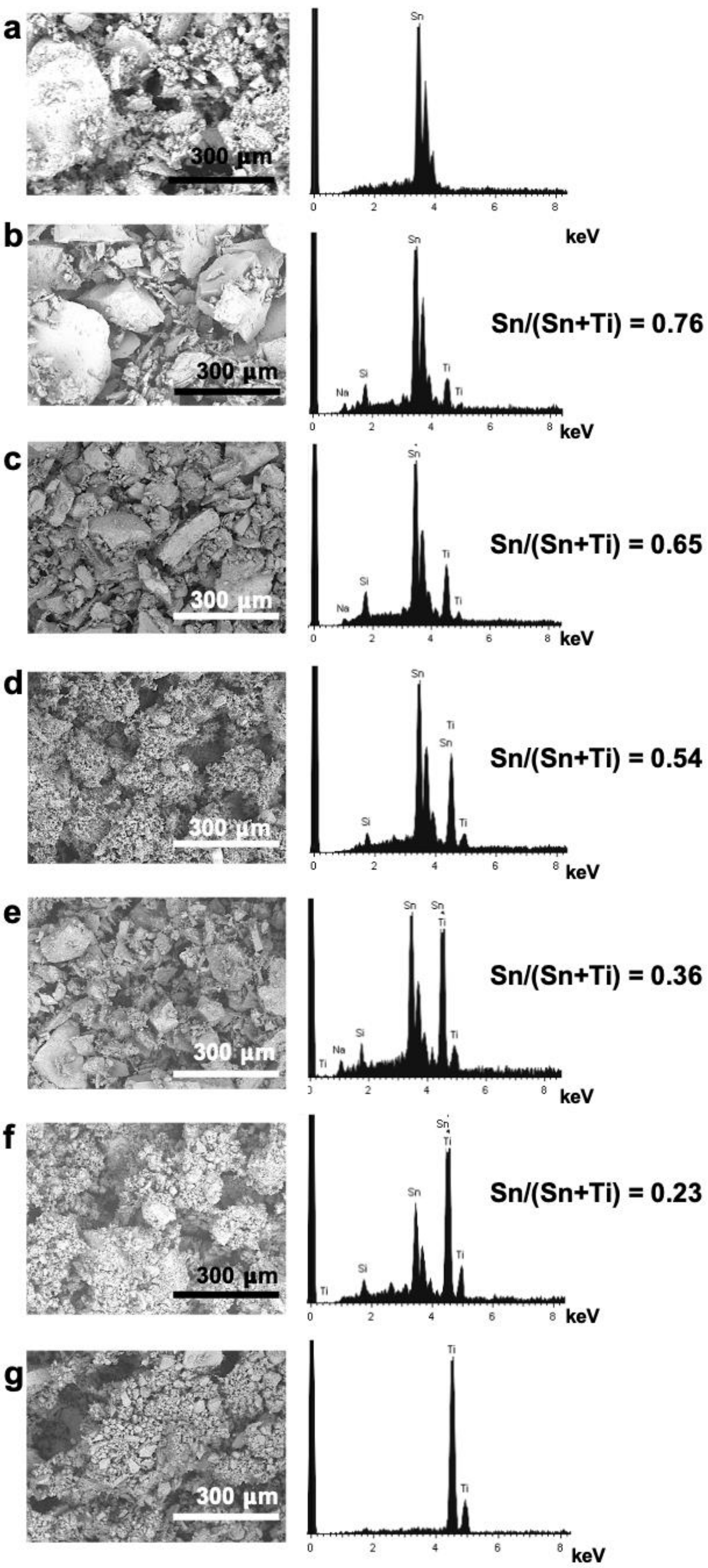

Figure S2. SEM images alongside with the relative EDX spectra of (**a**) 32:1 SnO_2/GO, (**b**) 32:1 $Sn_{0.71}Ti_{0.29}O_2$/GO, (**c**) 32:1 $Sn_{0.55}Ti_{0.45}O_2$/GO, (**d**) 32:1 $Sn_{0.44}Ti_{0.56}O_2$/GO, (**e**) 32:1 $Sn_{0.35}Ti_{0.65}O_2$/GO, (**f**) 32:1 $Sn_{0.21}Ti_{0.79}O_2$/GO, (**g**) 32:1 TiO_2/GO. The molar ratios Sn/(Sn+Ti) by EDX analysis have been reported, accordingly.

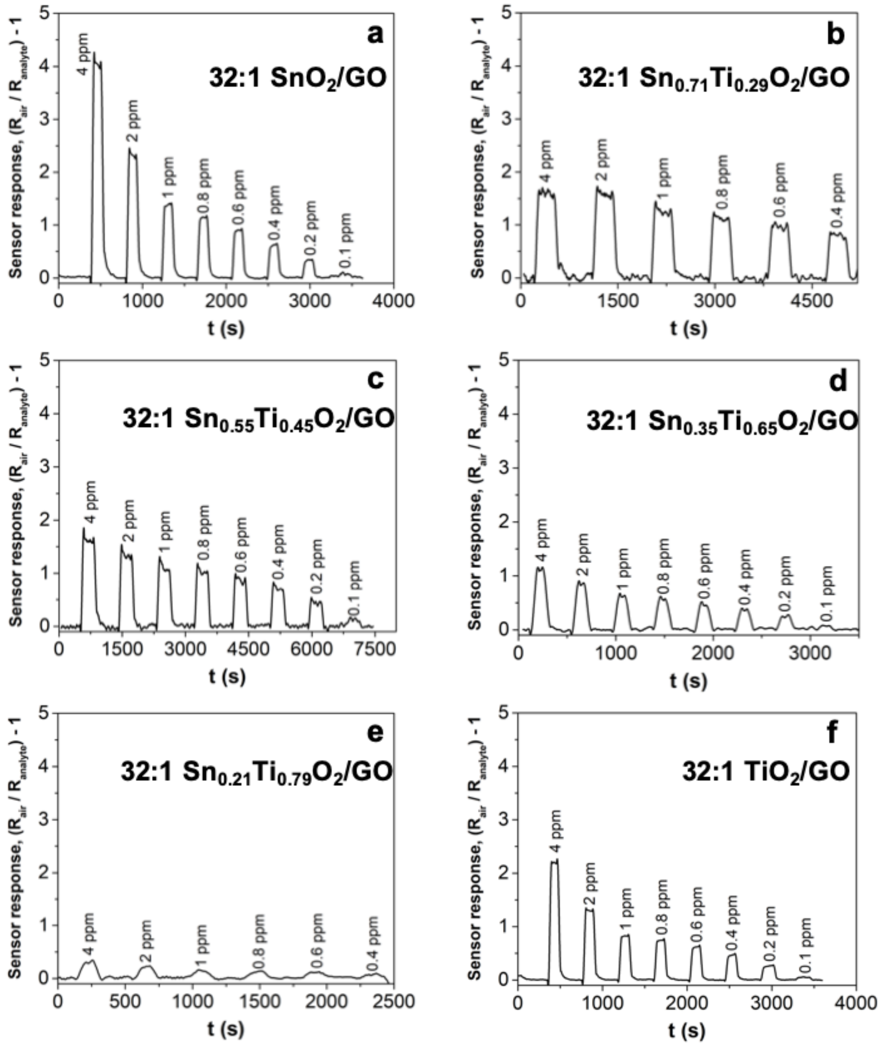

Figure S3. Toluene sensing by (**a**) 32:1 SnO_2/GO, (**b**–**e**) mixed oxides (with the exception of 32:1 $Sn_{0.44}Ti_{0.56}O_2$/GO, since it did not show any signal), and (**f**) 32:1 TiO_2/GO compounds. Tests were performed at 350 °C, without UV light, in simulated air (20% O_2 – 80% N_2).
